# Olfactory Receptor Gene Regulation in Insects: Multiple Mechanisms for Singular Expression

**DOI:** 10.3389/fnins.2021.738088

**Published:** 2021-09-16

**Authors:** Kaan Mika, Richard Benton

**Affiliations:** Center for Integrative Genomics, Faculty of Biology and Medicine, University of Lausanne, Lausanne, Switzerland

**Keywords:** olfactory receptor, sensory neuron, gene expression, neurodevelopment, evolution, feedback, *Drosophila*, insects

## Abstract

The singular expression of insect olfactory receptors in specific populations of olfactory sensory neurons is fundamental to the encoding of odors in patterns of neuronal activity in the brain. How a receptor gene is selected, from among a large repertoire in the genome, to be expressed in a particular neuron is an outstanding question. Focusing on *Drosophila melanogaster*, where most investigations have been performed, but incorporating recent insights from other insect species, we review the multilevel regulatory mechanisms of olfactory receptor expression. We discuss how *cis*-regulatory elements, *trans*-acting factors, chromatin modifications, and feedback pathways collaborate to activate and maintain expression of the chosen receptor (and to suppress others), highlighting similarities and differences with the mechanisms underlying singular receptor expression in mammals. We also consider the plasticity of receptor regulation in response to environmental cues and internal state during the lifetime of an individual, as well as the evolution of novel expression patterns over longer timescales. Finally, we describe the mechanisms and potential significance of examples of receptor co-expression.

## Introduction

Most animals possess large families of olfactory receptors, which enable detection of diverse chemical signals in their environment. In insects, as in vertebrates, the majority of individual receptors are expressed in unique populations of olfactory sensory neurons (OSNs), a property critical for the representation of odor-evoked neural activity in the brain. How the specificity of insect receptor expression is defined has been an unresolved problem for two decades.

Early work, mainly in adult *Drosophila melanogaster*, focused on identifying *cis*-regulatory sequences of olfactory receptor genes as well as transcription factors (TFs) required to promote their correct expression [reviewed in Fuss and Ray (2009) and Barish and Volkan (2015)]. Here we discuss recent advances, in which new experimental approaches in *D. melanogaster* and other insect models reveal multiple levels by which selective olfactory receptor expression is achieved and the plasticity of these processes over short and long timescales. We also make select comparisons with receptor choice in mammals, which relies on a combination of stochastic and deterministic mechanisms (Dalton and Lomvardas, 2015; Monahan and Lomvardas, 2015), to illustrate convergent or divergent strategies to achieve singular receptor expression.

## Insect Olfactory System Basics

**FIGURE 1:**
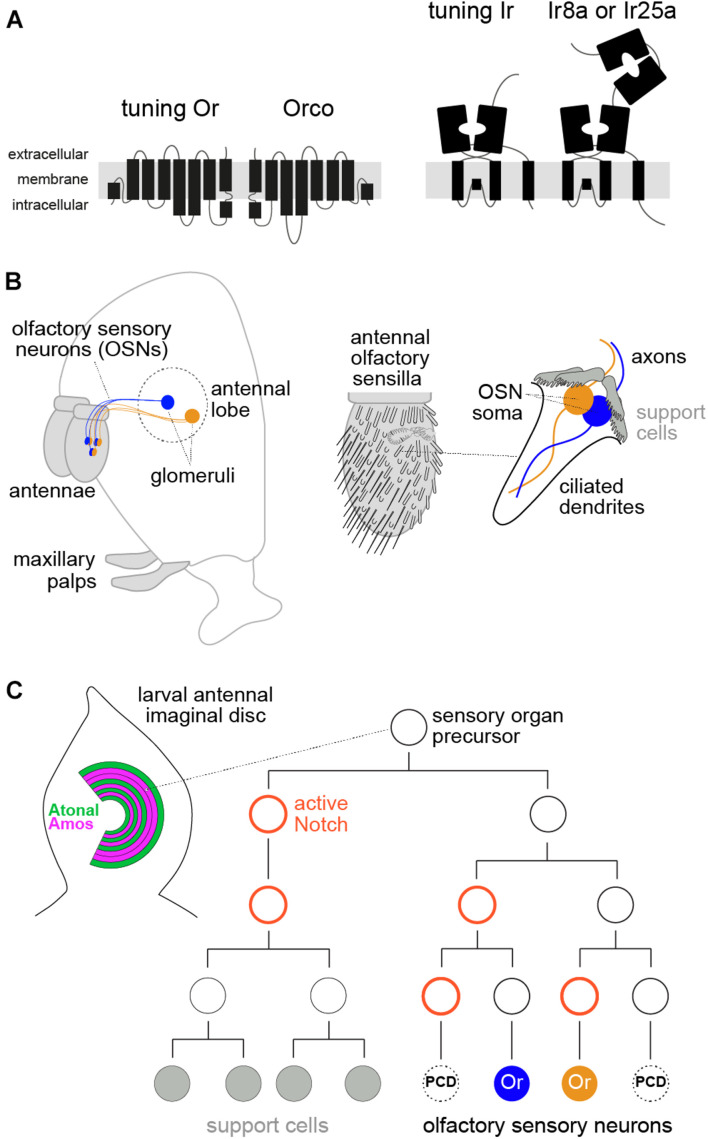
Molecular, anatomical, and developmental properties of the peripheral olfactory system in *D. melanogaster*. **(A)** Schematic of the two main insect olfactory receptor families. Odorant receptors (Ors) are seven transmembrane domain proteins that form heteromeric odor-gated ion channels composed of subunits of a ligand-specific (“tuning”) receptor and a co-receptor, Orco. Ionotropic receptors (Irs) are distantly related to ionotropic glutamate receptors, and function as odor-gated channel complexes composed of tuning Ir subunits and co-receptors (Ir8a or Ir25a). **(B)** Left: schematic of the *D. melanogaster* head (facing left) illustrating the main olfactory organs (antennae and maxillary palps, gray shading) and connectivity of two populations of olfactory sensory neurons (OSNs) to the antennal lobe in the brain. Right: schematic of the antenna, which is covered with diverse classes of sensory sensilla; the cellular organization of one sensillum, housing two OSNs, is shown on the far right (see text). **(C)** Left: schematic of the larval antennal imaginal disc, showing the concentric arcs of cells where different sensory organ precursors (SOP) are born. Amos- and Atonal-positive arcs give rise to OSN lineages expressing Ors and Irs, respectively, while other patterning determinants (not shown) are thought to specify SOP identity for different sensilla subtypes. Right: a simplified developmental lineage of an SOP producing a sensillum class with two OSNs. Two other potential neurons are removed by programmed cell death (PCD). Delta/Notch signaling determines the asymmetry of cell divisions, while many other patterning factors (not shown) are involved in specifying cell identity, encompassing both receptor expression and glomerular targeting of different OSNs (see text).

Insects have two main olfactory receptor families: odorant receptors (Ors) and ionotropic receptors (Irs) (Figure 1A). Both function as heteromeric odor-gated ion channels composed of subunits of a ligand-specific (“tuning”) receptor, which is expressed in a unique population of OSNs, and a broadly expressed, family-specific co-receptor (Orco for Ors; Ir8a or Ir25a for Irs) (Clyne et al., 1999b; Vosshall et al., 2000; Larsson et al., 2004; Couto et al., 2005; Nakagawa et al., 2005; Benton et al., 2006, 2009; Sato et al., 2008; Abuin et al., 2011; Butterwick et al., 2018; Del Marmol et al., 2021). *Or* and *Ir* genes are dispersed throughout insect genomes, but many occur in tandem arrays (Robertson et al., 2003; Gomez-Diaz et al., 2018), presumably reflecting their genesis by non-allelic homologous recombination.

Olfactory sensory neurons are housed in two olfactory organs in *D. melanogaster* (and other insects), the antenna and maxillary palp (Figure 1B). Each OSN extends a ciliated dendrite, where receptor proteins localize, into porous cuticular hairs on the organ surface (Schmidt and Benton, 2020; Gonzales et al., 2021). OSN axons project to the antennal lobe in the brain (Figure 1B). Neurons expressing the same olfactory receptor converge onto specific glomeruli, where they synapse with projection neurons that carry sensory information to higher brain centers (Grabe and Sachse, 2018; Schlegel et al., 2021). Each hair houses the dendrites of 1–4 OSNs, flanked by four support cells, which together comprise a sensillum. There are several distinct morphological classes of sensilla (Figure 1B), each of which has multiple subtypes characterized by a stereotyped number of OSNs and receptor expression profile.

Adult *D. melanogaster* has ∼2200 OSNs (within the two antennae and maxillary palps), encompassing ∼30 Or-expressing and ∼10 Ir-expressing classes (Couto et al., 2005; Benton et al., 2009; Grabe et al., 2016). This complexity is roughly one-to-several orders of magnitude lower than presumed OSN types in mammals, based upon receptor numbers (Hughes et al., 2018). Some other insect species, notably ants, have several hundred *Or*s (Yan et al., 2020).

## Insect Olfactory System Development

Olfactory receptor expression must be appreciated in the context of OSN development. Sensilla arise from sensory organ precursors (SOPs), which are specified within a set of concentric arcs in the larval antennal imaginal disc (Rodrigues and Hummel, 2008; Barish and Volkan, 2015; Yan et al., 2020; Figure 1C). During early pupal stages, each SOP gives rise to a short lineage of three rounds of cell division, to produce four support cells and, potentially, four OSNs. However, up to three of these neuron precursors (depending upon the sensillum class) are removed by precisely patterned programmed cell death, yielding the final set of OSNs (Endo et al., 2007, 2011; Barish and Volkan, 2015; Chai et al., 2019; Prieto-Godino et al., 2020; Figure 1C).

The sensillum class a given SOP will produce is determined in the antennal disc by spatially restricted TFs, including Amos and Atonal, which demarcate Or and Ir OSN precursors, respectively (Figure 1C), and Dachshund and Rotund; these proteins all exhibit zonally restricted expression (or form gradients) across the rings where olfactory SOPs are specified (Rodrigues and Hummel, 2008; Barish and Volkan, 2015; Chai et al., 2019; Yan et al., 2020). Individual SOP classes therefore likely have a unique molecular identity before initiating cell division, though this has not been characterized. Each division is asymmetric, determined by Notch/Delta signaling (Figure 1C), to give rise to daughter cells of unique identity (Endo et al., 2007, 2011). The terminal cells of the neuronal sub-lineage are presumed to have a distinct set of fate determinants that specify the expression of receptors (Endo et al., 2007, 2011; Li et al., 2013; Barish and Volkan, 2015; Chai et al., 2019), but the molecular profile of these early developmental stages is still incompletely understood.

## Olfactory Receptor Spatio-Temporal Expression

Knowledge of the timing of olfactory receptor expression is critical to distinguish if developmental regulators have direct or indirect roles in inducing receptor gene transcription. Recent antennal bulk and single-cell/nuclear OSN RNA-sequencing at multiple timepoints indicates that transcripts for a subset of receptors are first detected from ∼24 h after puparium formation (Pan et al., 2017; Li et al., 2020; McLaughlin et al., 2021), at most a few hours after the terminal division of these lineages (Endo et al., 2011; Chai et al., 2019). Other receptors initiate expression over the subsequent ∼24–48 h, potentially reflecting asynchrony in SOP lineage development and/or differences in the mechanisms/levels of transcriptional induction. Most importantly, the single-OSN transcriptomes indicate the vast majority of individual OSNs express only one receptor gene from the earliest stages of the process. This contrasts with *OR* expression in mice, where immature OSNs transiently express low levels of multiple receptors before a single gene is chosen for high-level transcription (Hanchate et al., 2015; Tan et al., 2015). Furthermore, unlike the monoallelic *OR* expression observed in mammals (Monahan and Lomvardas, 2015), endogenous gene-tagging indicates that both receptor alleles are expressed in insect OSNs (Kurtovic et al., 2007; Grosjean et al., 2011; Auer et al., 2020).

The onset of receptor expression occurs in parallel with, or after, OSN axons converge on glomeruli in the antennal lobe (Jefferis et al., 2004; Jefferis and Hummel, 2006; Li et al., 2021). This timing is consistent with the lack of contributions of receptors to neuronal guidance (Dobritsa et al., 2003), in contrast to mammalian ORs, which have an important, though indirect, role in regulating glomerular convergence of OSNs (Sakano, 2010). However, antennal developmental transcriptomics in the clonal raider ant, *Ooceraea biroi*, revealed that receptors are expressed prior to glomerulus formation (Ryba et al., 2020), with genetic evidence hinting that Orco (at least) contributes during development to formation or maintenance of these structures (Trible et al., 2017; Yan et al., 2017; Ryba et al., 2020). In adult *D. melanogaster*, receptor transcripts continue to accumulate several days after eclosion before levels plateau (Jafari et al., 2021), indicating the continuity and/or maturation of mechanisms inducing their expression.

## *Cis*-Regulatory Elements

**FIGURE 2:**
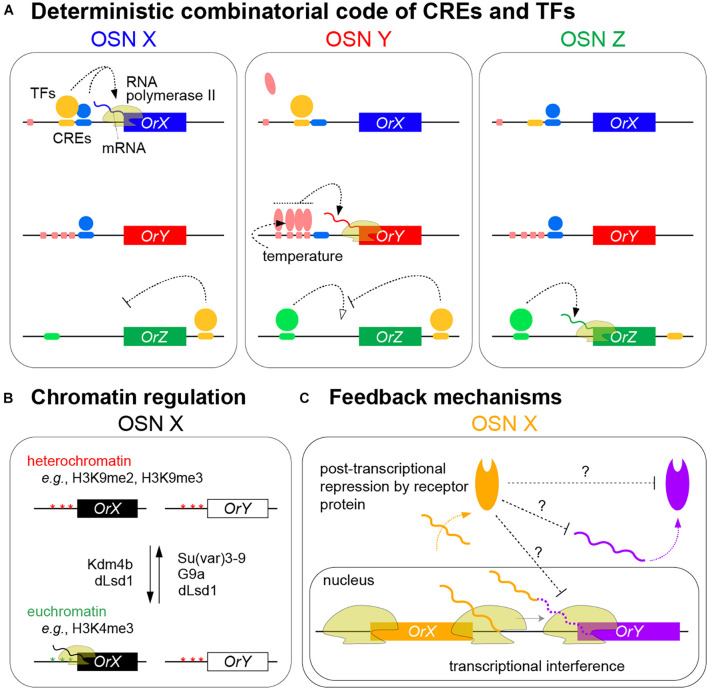
Models of olfactory receptor expression in insects. **(A)** Summary of the mechanisms ensuring the neuron-specific transcription of olfactory receptors through the combinatorial action of CREs and TFs to promote RNA polymerase II transcription of a specific receptor gene in an olfactory sensory neuron (OSN) (only the neuronal nuclei are shown). In these hypothetical examples, *OrX* requires binding of both yellow and blue TFs to corresponding CREs to be expressed; either alone is insufficient. *OrY* requires the cooperative binding of the red TF to clustered CREs for expression; this cooperation can ensure robust expression in the face of environmental temperature changes; by contrast, the red TF does not bind to the single corresponding CRE upstream of *OrX* in these neurons. *OrZ* transcription is promoted by the green TF but suppressed by the yellow TF that binds 3′ of the gene. Other external factors might influence levels, though not spatial patterning, of receptor expression (see text). **(B)** Chromatin marks and histone-modifying enzymes contributing to the selective expression of olfactory receptors. Different enzymes display differences in their temporal expression and requirement; among these, dLsd1 – which is normally associated with removing H3K4 methylation – appears to have roles in OSNs in both promoting and repressing *Or* expression (see text). Although schematized separately for clarity, chromatin regulation is intimately related to the combinatorial binding of TFs to receptor loci. **(C)** Feedback mechanisms contributing to the refinement and/or stability of receptor expression. Transcriptional interference by *OrX* of *OrY* might occur when inefficient transcriptional termination at the 3′ end of the former gene leads to the RNA polymerase II impeding transcription initiation at *OrY* (solid wavy orange and purple lines represent protein coding transcripts from *OrX* and *OrY*, respectively; the dashed purple line represents the 3′UTR of *OrX* transcripts that incorporate sequences encoded by *OrY* that are not translated into OrY) (Mika et al., 2021). Receptor protein-dependent feedback on transcript or protein levels of other (not necessarily closely linked) receptors occurs through unknown mechanisms (Maguire et al., 2020; Jafari et al., 2021; Mika et al., 2021).

The genetically hardwired and stable choice of receptor transcription in OSNs has promoted extensive efforts to define *cis*-regulatory elements (CREs) of receptor genes through bioinformatic identification of DNA motifs (e.g., by phylogenetic footprinting) and experimental “enhancer bashing” (Ray et al., 2007, 2008; Miller and Carlson, 2010; Silbering et al., 2011; Prieto-Godino et al., 2017). These efforts – reviewed extensively elsewhere (Fuss and Ray, 2009; Barish and Volkan, 2015; Yan et al., 2020) – have revealed that CREs defining correct OSN expression are generally encompassed within a few 100–1000 base pairs upstream of coding sequences, although 3′ and intronic regions are important for certain genes. Some CREs are necessary to promote expression, while others prevent expression in inappropriate cell types. There is no evidence for distantly acting regulatory elements of insect receptor genes – as identified in some tandem arrays of mammalian receptor genes (Monahan and Lomvardas, 2015) – although clustered insect genes might share common regulatory sequences (Prieto-Godino et al., 2017). Detailed dissection of specific *Or* promoters further illustrates how the order, number, and overlap of individual CREs are critical for defining robust and selective receptor expression (Jafari and Alenius, 2015; Gonzalez et al., 2019). These advances support a model in which unique combinations of locally acting CREs ensure the correct transcriptional activation in (and only in) a given class of OSNs (Figure 2A). However, our global understanding of *cis*-regulation remains fragmentary: only a subset of CREs within larger genomic fragments have been identified for a few receptors and only a subset of these CREs have known binding proteins.

## *Trans*-Acting Factors

Several TFs required for the correct expression of receptor genes in specific populations of neurons have been identified in *D. melanogaster* through loss-of-function genetic screens (Jafari et al., 2012; Chai et al., 2019; Mika et al., 2021), candidate approaches (Tichy et al., 2008; Li et al., 2013), and expression screens (Li et al., 2020) [reviewed in Fuss and Ray (2009); Barish and Volkan (2015), and Yan et al. (2020)]. Analogous to contributions of CREs, TFs can promote or repress receptor expression (and can have different roles for different genes), and unique combinations of these factors are required for individual receptors (Figure 2A). The convergence of several genetic screens on the same TFs (e.g., Pdm3 and E93) (Jafari et al., 2012; Chai et al., 2019; Mika et al., 2021) suggests that a majority of the core *trans*-acting regulatory proteins have been identified. These TFs contain diverse types of DNA binding domains and while some orthologous proteins might have similar roles in other insects (e.g., Acj6) (Clyne et al., 1999a; Fujii et al., 2011), they are not obviously related to key TFs functioning in *OR* expression in mice (Dalton and Lomvardas, 2015; Monahan and Lomvardas, 2015). In *D. melanogaster*, this core set is theoretically more than adequate in number (∼15–20) to contribute combinatorially to a unique gene regulatory network within each OSN class.

Despite this conceptual framework, many issues remain unresolved. Only a subset of TFs have defined binding motifs, and even fewer have been shown to associate physically with receptor gene regulatory sequences (typically in *in vitro* assays) (Bai et al., 2009; Jafari et al., 2012). Moreover, the presence of a motif in a CRE for a given gene does not necessarily mean that the corresponding TF is required (and vice versa) (Jafari et al., 2012; Figure 2A). While some TFs have lineage-specific expression and function (Li et al., 2020; Arguello et al., 2021), many have broad expression in OSNs despite very selective requirements in receptor regulation (Jafari et al., 2012; Li et al., 2020). The lack of correlation between the presence of a TF binding motif in a CRE and TF requirement for a given receptor might reflect differences in *in vitro* and *in vivo* binding specificities for TFs and/or an indirect requirement for *trans*-acting factors in controlling receptor expression. Indeed, temporal manipulation of TF function indicates that several of these proteins have multiple roles in OSN development, for example, during SOP lineage specification (Bai and Carlson, 2010; Jafari et al., 2012; Chai et al., 2019; Arguello et al., 2021; Mika et al., 2021). Moreover, many TFs are expressed and required in late pupal/adult stages implying roles in both initiation and maintenance of correct receptor expression (Bai and Carlson, 2010; Jafari et al., 2012; Arguello et al., 2021; Mika et al., 2021). The biochemical properties of TF/CRE interactions that promote stable receptor expression in a given OSN type remain, however, largely elusive.

## Chromatin Marks and Chromosomal Interactions

Recent genetic screens and candidate analyses have also identified roles for various chromatin modifiers (e.g., histone methyltransferases and deacetylases or their regulators) in the correct activation and/or repression of receptor genes (Sim et al., 2012; Alkhori et al., 2014; Jafari and Alenius, 2015; Chai et al., 2019; Gonzalez et al., 2019; Jafari et al., 2021; Figure 2B). Conserved epigenetic modifications, such as H3K4me3 and H3K9me2 – normally associated with active and repressed promoters, respectively – have been detected at individual receptor genes by chromatin immunoprecipitation (ChIP)-quantitative RT-PCR (Sim et al., 2012; Alkhori et al., 2014; Jafari and Alenius, 2015; Gonzalez et al., 2019; Jafari et al., 2021). Temporal analyses of the expression and requirement for some of these enzymes have begun to reveal different phases in how chromatin modifications may impact receptor expression, focusing on an *Or59b* promoter transgenic reporter (Jafari and Alenius, 2015; Gonzalez et al., 2019; Jafari et al., 2021). The H3K9me3 demethylase Kdm4B participates in the initiation of reporter expression, while Su(var)3–9 – which promotes H3K9me3 and heterochromatin formation – helps prevent ectopic expression. The activity of Su(var)3–9 appears to be antagonized by dLsd1, which contributes to reporter expression throughout OSN development. The activating role of dLsd1 in OSNs is intriguing as in other *D. melanogaster* tissues this enzyme erases H3K4 methylation to induce heterochromatin formation; this olfactory function highlights a potential parallel with mammalian Lsd1 function in facilitating *OR* expression (Figure 2B; Dalton and Lomvardas, 2015; Monahan and Lomvardas, 2015). Su(var)3–9 and dLsd1 expression increases after hatching and have been proposed to contribute to the termination of a “critical period” of receptor expression in young adults when the mature pattern is stabilized (Jafari et al., 2021).

Despite these insights, a global time course of chromatin state at active and silenced endogenous receptor loci in specific neuron populations is lacking, constrained by the ability to obtain enough cells of a given class for ChIP-sequencing-based methods. A low-resolution assessment of chromatin structure in several individual mature *Ir*-expressing OSN populations has been made using Chromatin Accessibility Targeted DamID (CATaDa) (Arguello et al., 2021), which exploits cell-type specific expression of the *E. coli* Dam methylase to avoid a need for cell sorting (Aughey et al., 2018). This analysis revealed that access to the DNA at different receptor genes is globally similar between neuron populations, suggesting that the specificity of transcriptional activation in a given neuron is not reliant upon uniquely accessible enhancers (at least in the analyzed *Ir* populations) (Arguello et al., 2021). Although direct comparison is currently hard, this situation might contrast with that in mammals, where all but the chosen receptor gene are maintained in a heterochromatic, silenced state (Dalton and Lomvardas, 2015; Monahan and Lomvardas, 2015). In mice, higher-level structural properties of DNA, notably interchromosomal interactions and nuclear compartmentalization of olfactory receptor genes, are important for the expression of one receptor allele and silencing of all others (Bashkirova and Lomvardas, 2019; Monahan et al., 2019), but whether such phenomena are important in insect OSNs is unknown.

## Feedback Mechanisms

A central mechanism ensuring singular receptor expression in mammals is a feedback signal from the chosen receptor (Dalton and Lomvardas, 2015; Monahan and Lomvardas, 2015). Intriguingly, this feedback pathway has co-opted the unfolded protein response, through which the expressed OR induces translational homeostasis in OSNs to, ultimately, stabilize *OR* choice and prevent activation of other receptor genes (Dalton et al., 2013). In insects, feedback mechanisms were thought not to exist, as receptor genes can be ectopically expressed in other OSNs without affecting endogenous receptor gene expression (e.g., Ray et al., 2007), and neurons lacking their own receptors (through mutation) do not appear to activate expression of other receptor loci (e.g., Dobritsa et al., 2003; Grosjean et al., 2011).

Recent evidence, however, supports the existence of regulatory relationships between some receptor genes that might help to reinforce the singular expression of receptors defined by OSN-specific TF combinations (Figure 2C). In a tandem array of *D. melanogaster* genes (*Ir75c*, *Ir75b*, and *Ir75a*), transcription from the upstream genes was found to run through the downstream genes, blocking their expression in *cis*, potentially through transcriptional interference (Mika et al., 2021). Ir75c can also prevent accumulation of the other receptor proteins in *trans*, through a protein-dependent, post-transcriptional (but unknown) mechanism (Mika et al., 2021; Figure 2C). Whether similar interactions occur between other clustered genes is unclear, but such phenomena might help explain how recent receptor duplicates initially acquire exclusive expression patterns. In the mosquito, *Anopheles gambiae*, broad transgenic overexpression of one *Or* led to reduced transcription of most other *Or*s, but not the vast majority of other OSN-expressed genes (Maguire et al., 2020). This suppression mechanism is also unknown, but appears to depend upon the ectopically expressed Or protein (Figure 2C). Similar transcriptional suppression of *Or*s upon widespread misexpression of one receptor was also reported in *D. melanogaster* (Jafari et al., 2021). In either species, it is unclear whether this type of repression uses a similar or different pathway to mammalian OR feedback, and if such a pathway operates downstream of endogenously expressed, and not only transgenically expressed, receptors.

## Environmental and Internal State Influences

Although the precise spatial patterning of receptor expression is under the control of hard-wired genetic programs, growing evidence indicates that an animal’s internal state and environmental cues can impact the level of receptor expression, facilitated by the ease of performing RNA-sequencing in diverse species under different conditions. For example, the mating status of *Drosophila suzukii* and the pine caterpillar moth, *Dendrolimus punctatus*, are linked to changes in expression of some *Or*s (Zhang et al., 2017; Crava et al., 2019). Blood-feeding in mosquitoes leads to transcriptional down- or up-regulation of certain olfactory receptors (Rinker et al., 2013; Matthews et al., 2016). Odor exposure itself can lead to changes in receptor expression in *D. melanogaster* (Zhou et al., 2009; von der Weid et al., 2015; Koerte et al., 2018) although the affected receptors are not necessarily those that respond to the odor stimulus (Koerte et al., 2018). Similarly, in the honeybee, *Apis mellifera*, olfactory conditioning can cause alterations in receptor expression (Claudianos et al., 2014).

In most of these examples, we know little about the physiological and ecological significance of such changes or how external factors influence receptor expression. However, analysis of the impact of temperature stress and starvation upon the transcription of endogenous receptors and transgenic reporters in *D. melanogaster* has revealed the importance of cooperation between clustered CREs to buffer against environment fluctuations, hinting at a biochemical basis ensuring robust receptor expression (Jafari and Alenius, 2015; Gonzalez et al., 2019; Jafari et al., 2021). Temperature stress also affects the expression of chromatin modifying enzymes, which might contribute to the stabilization of ectopic reporter expression (Jafari et al., 2021). Further study of such short-term plasticity of receptor expression might help reveal new insights into the mechanisms that promote their selective neuronal expression.

## Evolvability

The overall precision of olfactory receptor expression within a species belies the flexibility of this sensory system over evolutionary timescales (Ramdya and Benton, 2010). Comparative antennal transcriptomic studies (using bulk RNA-sequencing) in closely related species have revealed differences in expression level of many receptors (McBride et al., 2014; Shiao et al., 2015; Crowley-Gall et al., 2016; Pan et al., 2017), although these datasets cannot distinguish changes in receptor expression level within an OSN population from changes in numbers of neurons expressing a particular gene. More strikingly, enormous variation exists in the size of olfactory receptor repertoires (from <10 to >500) – and, presumably, corresponding number of neuron types – between species (Robertson, 2019; Yan et al., 2020).

How new olfactory receptor expression patterns evolve to define a distinct neuron class is largely obscure. Even relatively recently duplicated receptor genes can have quite different *cis*- and *trans*-regulatory mechanisms (Prieto-Godino et al., 2017; Mika et al., 2021), prohibiting easy identification of the responsible genetic changes that drove the divergence in their spatial expression. The evolution of new receptor expression patterns is of course intimately linked with the evolution of novel neuron types. One potential way new OSN classes can be created is through changes in the genetically patterned programmed cell death that normally removes many populations during development (Sen et al., 2004; Endo et al., 2007; Chai et al., 2019; Figure 1C). Artificial blockage of programmed cell death in the developing sensory lineages in *D. melanogaster* is sufficient to generate “undead” neurons that express olfactory receptors (Prieto-Godino et al., 2020). Intriguingly, the subset of receptor genes transcribed in undead neurons is enriched for those that are found in tandem arrays, and which are (exceptionally) co-expressed in “normal” OSNs (see below). The reason for this phenomenon is unknown but hints at a molecular property of these tandem arrays (e.g., chromatin state) that makes one or more of the constituent receptor genes permissive for expression in OSN precursors that are normally condemned to die.

## Receptor Co-Expression

While we have emphasized mechanisms underlying the discrete expression of olfactory receptors, there are cases of receptor co-expression. The most obvious examples are co-expression of tuning receptors with co-receptor subunits (Larsson et al., 2004; Nakagawa et al., 2005; Benton et al., 2006; Sato et al., 2008; Abuin et al., 2011; Figure 1A). The mechanisms specifying the broad expression of co-receptors are mostly unknown (Mika et al., 2021) and these genes might use different gene regulatory networks to those of tuning receptors. Analysis in *D. melanogaster* and the mosquito *Aedes aegypti* showed that different co-receptors are not mutually exclusive, and can often be detected in OSN classes where they do not have a (known) partner tuning receptor (Abuin et al., 2011; Task et al., 2020; Younger et al., 2020). These observations raise the interesting possibility that some neurons have two types of receptors contributing to their response profile (Younger et al., 2020) and/or that co-receptors alone modulate the responses of other receptor classes (Task et al., 2020; Vulpe et al., 2021). Alternatively, overlapping co-receptor expression might simply reflect a lack of regulatory pathways to constrain their broad expression to neurons in which they function.

Several examples of co-expressed tuning receptors have been described in various insect species. In some cases, two receptors arise from alternative splicing of transcripts expressed from a common locus (Robertson et al., 2003; Ray et al., 2007; Lebreton et al., 2017). Other examples of co-expression appear to be due to di/polycistronic transcripts encoded by clustered receptor genes (Ray et al., 2007; Koutroumpa et al., 2014; Karner et al., 2015). However, caution is necessary in interpretation of such “co-expression” based upon RNA *in situ* hybridization data alone, because this can be confounded by the existence of read-through transcription, where exons of downstream genes in tandem arrays are incorporated into the transcripts of upstream genes, but not encode the corresponding receptor protein (Prieto-Godino et al., 2017; Mika et al., 2021). Notably, in *A. aegypti*, the number of tuning receptors expressed in olfactory organs (determined by bulk RNA-sequencing) is in large excess of the number of glomeruli, suggesting that co-expression of tuning receptors is widespread in this insect (Younger et al., 2020).

There are still only a few clear examples of co-expressed tuning receptor genes that encode functionally distinct proteins. Some of these genes are adjacent in the genome, consistent with conservation of CREs upon gene duplication (Dobritsa et al., 2003), while others are unlinked (Goldman et al., 2005), suggesting convergence in their *cis*-regulatory landscape. Tuning receptor co-expression can expand the response profile of a neuron class (Lebreton et al., 2017), although in many cases it might reflect a “transient” evolutionary state where duplicated receptor genes have not yet acquired distinct expression patterns (Ramdya and Benton, 2010).

## Discussion

The exquisite specificity of insect olfactory receptor expression is widely viewed as resulting from a deterministic process relying on sets of TFs acting through receptor-gene specific combinations of CREs (Ray et al., 2007). While this model remains largely valid, two issues require further investigation.

First, our knowledge of the molecular biology of receptor choice is still superficial: we do not have a complete picture of the CREs, the chromatin state, and the associated TFs for any receptor gene. Such properties are extraordinarily hard to characterize in insect OSNs, given their rarity, small size, and difficulty to extract them from (or image them within) cuticle-covered tissues, as well as the relatively rapid development from SOP to mature neuron. However, new *in vivo* cell-type specific RNA/chromatin profiling and transgenesis-based approaches in *D. melanogaster* (and, in theory, in other genetically manipulatable species) (van den Ameele et al., 2019; Li, 2020) might aid in better understanding these mechanistic details. The relatively compact size of most receptor gene regulatory elements – in comparison to many other neural gene enhancers – suggests that the problem is tractable, and further study could offer general insights into how genes exhibit highly selective expression patterns in the nervous system.

Second, it is increasingly unclear to what extent receptor expression relies solely on a combinatorial code of CREs and TFs in all insects. This model was developed principally from studies in *D. melanogaster*, where the receptor repertoires might be sufficiently small to be regulated by deterministic processes. However, there is growing evidence for feedback mechanisms and dynamic chromatin regulation in this insect, as well as hints that species with larger receptor repertoires use additional/alternative regulatory mechanisms. These advances raise the possibility that greater mechanistic similarities – or at least analogies – exist with the process of olfactory receptor choice in mammals than currently appreciated.

## Author Contributions

KM and RB wrote the manuscript. Both authors contributed to the article and approved the submitted version.

## Conflict of Interest

The authors declare that the research was conducted in the absence of any commercial or financial relationships that could be construed as a potential conflict of interest.

## Publisher’s Note

All claims expressed in this article are solely those of the authors and do not necessarily represent those of their affiliated organizations, or those of the publisher, the editors and the reviewers. Any product that may be evaluated in this article, or claim that may be made by its manufacturer, is not guaranteed or endorsed by the publisher.
